# Supplementary material for: Resolving the full spectrum of human genome variation using Linked-Reads
Source: Genome Res. 2019 Apr;29(4):635–45. doi: 10.1101/gr.234443.118 (PMC6442396; doi:10.1101/gr.234443.118)
Supplement: Supplemental Material [file supp_gr.234443.118_Supplemental_longranger-2.2.zip › longranger-2.2.tar.bz2!/lr2.2-public/lib/go/src/code.google.com/vitess/docs/overview/concepts.html]

Vitess / Key Concepts


Toggle navigation

- Overview
- Guides
- Reference
- Blog
- GitHub

- #### Overview

  - What is Vitess
  - Scaling MySQL with Vitess
  - Key Concepts
- #### Getting Started

  - Run Vitess on Kubernetes
    - Custom Docker Build
  - Run Vitess Locally
- #### User Guide

  - Introduction- Client Libraries- Backing Up Data- Reparenting
        - Schema Management
        - VSchema Guide
        - Schema Swap (Tutorial)
        - Sharding
          - Horizontal Sharding (Tutorial, automated)
          - Horizontal Sharding (Tutorial, manual)
          - Sharding in Kubernetes (Tutorial, automated)
          - Sharding in Kubernetes (Tutorial, manual)
        - Vitess Sequences
        - MySQL Server Protocol
        - Vitess and Replication
        - Update Stream
        - Row Based Replication
        - Topology Service
        - Transport Security Model
        - Launching
          - Scalability Philosophy
          - Production Planning
          - Server Configuration
          - 2PC Guide
          - Troubleshooting
        - Upgrading
- #### Reference Guides

  - Vitess API- vtctl Commands
- #### Other Resources

  - Presentations- Blog- Roadmap
- #### Contributing

  - Contributing to Vitess- GitHub Workflow- Code Reviews
- GitHub

# Key Concepts

- #### Overview

  - What is Vitess
  - Scaling MySQL with Vitess
  - Key Concepts
- #### Getting Started

  - Run Vitess on Kubernetes
    - Custom Docker Build
  - Run Vitess Locally
- #### User Guide

  - Introduction- Client Libraries- Backing Up Data- Reparenting
        - Schema Management
        - VSchema Guide
        - Schema Swap (Tutorial)
        - Sharding
          - Horizontal Sharding (Tutorial, automated)
          - Horizontal Sharding (Tutorial, manual)
          - Sharding in Kubernetes (Tutorial, automated)
          - Sharding in Kubernetes (Tutorial, manual)
        - Vitess Sequences
        - MySQL Server Protocol
        - Vitess and Replication
        - Update Stream
        - Row Based Replication
        - Topology Service
        - Transport Security Model
        - Launching
          - Scalability Philosophy
          - Production Planning
          - Server Configuration
          - 2PC Guide
          - Troubleshooting
        - Upgrading
- #### Reference Guides

  - Vitess API- vtctl Commands
- #### Other Resources

  - Presentations- Blog- Roadmap
- #### Contributing

  - Contributing to Vitess- GitHub Workflow- Code Reviews

This document defines common Vitess concepts and terminology.

## Keyspace

A *keyspace* is a logical database. In the unsharded case, it maps directly
to a MySQL database name, but it can also map to multiple MySQL databases.

Reading data from a keyspace is like reading from a MySQL database. However,
depending on the consistency requirements of the read operation, Vitess
might fetch the data from a master database or from a replica. By routing
each query to the appropriate database, Vitess allows your code to be
structured as if it were reading from a single MySQL database.

When a database is
sharded,
a keyspace maps to multiple MySQL databases. In that case, a single query sent
to Vitess will be routed to one or more shards, depending on where the requested
data resides.

## Keyspace ID

The *keyspace ID* is the value that is used to decide on which shard a given
record lives. Range-based Sharding
refers to creating shards that each cover a particular range of keyspace IDs.

Often, the keyspace ID is computed as the hash of some column in your data,
such as the user ID. This would result in randomly spreading users across
the range-based shards.
Using this technique means you can split a given shard by replacing it with two
or more new shards that combine to cover the original range of keyspace IDs,
without having to move any records in other shards.

Previously, our resharding process required each table to store this value as a
`keyspace_id` column because it was computed by the application. However, this
column is no longer necessary when you allow VTGate to compute the keyspace ID
for you, for example by using a `hash` vindex.

## Shard

A *shard* is a division within a keyspace. A shard typically contains one MySQL
master and many MySQL slaves.

Each MySQL instance within a shard has the same data (excepting some replication
lag). The slaves can serve read-only traffic (with eventual consistency guarantees),
execute long-running data analysis tools, or perform administrative tasks
(backup, restore, diff, etc.).

A keyspace that does not use sharding effectively has one shard.
Vitess names the shard `0` by convention. When sharded, a keyspace has `N`
shards with non-overlapping data.

### Resharding

Vitess supports dynamic resharding,
in which the number of shards is changed on a live cluster. This can be either
splitting one or more shards into smaller pieces, or merging neighboring shards
into bigger pieces.

During dynamic resharding, the data in the source shards is copied into the
destination shards, allowed to catch up on replication, and then compared
against the original to ensure data integrity. Then the live serving
infrastructure is shifted to the destination shards, and the source shards are
deleted.

## Tablet

A *tablet* is a combination of a `mysqld` process and a corresponding `vttablet`
process, usually running on the same machine.

Each tablet is assigned a *tablet type*, which specifies what role it currently
performs.

### Tablet Types

- **master** - A *replica* tablet that happens to currently be the MySQL master
  for its shard.
- **replica** - A MySQL slave that is eligible to be promoted to *master*.
  Conventionally, these are reserved for serving live, user-facing
  requests (like from the website's frontend).
- **rdonly** - A MySQL slave that cannot be promoted to *master*.
  Conventionally, these are used for background processing jobs,
  such as taking backups, dumping data to other systems, heavy
  analytical queries, MapReduce, and resharding.
- **backup** - A tablet that has stopped replication at a consistent snapshot,
  so it can upload a new backup for its shard. After it finishes,
  it will resume replication and return to its previous type.
- **restore** - A tablet that has started up with no data, and is in the process
  of restoring itself from the latest backup. After it finishes,
  it will begin replicating at the GTID position of the backup,
  and become either *replica* or *rdonly*.
- **drained** - A tablet that has been reserved by a Vitess background
  process (such as rdonly tablets for resharding).

## Keyspace Graph

The *keyspace graph* allows Vitess to decide which set of shards to use for a
given keyspace, cell, and tablet type.

### Partitions

During horizontal resharding (splitting or merging shards), there can be shards
with overlapping key ranges. For example, the source shard of a split may serve
`c0-d0` while its destination shards serve `c0-c8` and `c8-d0` respectively.

Since these shards need to exist simultaneously during the migration,
the keyspace graph maintains a list (called a *partitioning* or just a *partition*)
of shards whose ranges cover all possible keyspace ID values, while being
non-overlapping and contiguous. Shards can be moved in and out of this list to
determine whether they are active.

The keyspace graph stores a separate partitioning for each `(cell, tablet type)` pair.
This allows migrations to proceed in phases: first migrate *rdonly* and
*replica* requests, one cell at a time, and finally migrate *master* requests.

### Served From

During vertical resharding (moving tables out from one keyspace to form a new
keyspace), there can be multiple keyspaces that contain the same table.

Since these multiple copies of the table need to exist simultaneously during
the migration, the keyspace graph supports keyspace redirects, called
`ServedFrom` records. That enables a migration flow like this:

1. Create `new_keyspace` and set its `ServedFrom` to point to `old_keyspace`.
2. Update the app to look for the tables to be moved in `new_keyspace`.
   Vitess will automatically redirect these requests to `old_keyspace`.
3. Perform a vertical split clone to copy data to the new keyspace and start
   filtered replication.
4. Remove the `ServedFrom` redirect to begin actually serving from `new_keyspace`.
5. Drop the now unused copies of the tables from `old_keyspace`.

There can be a different `ServedFrom` record for each `(cell, tablet type)` pair.
This allows migrations to proceed in phases: first migrate *rdonly* and
*replica* requests, one cell at a time, and finally migrate *master* requests.

## Replication Graph

The *replication graph* identifies the relationships between master
databases and their respective replicas. During a master failover,
the replication graph enables Vitess to point all existing replicas
to a newly designated master database so that replication can continue.

## Topology Service

The *Topology Service*
is a set of backend processes running on different servers.
Those servers store topology data and provide a distributed locking service.

Vitess uses a plug-in system to support various backends for storing topology
data, which are assumed to provide a distributed, consistent key-value store.
By default, our local example
uses the ZooKeeper plugin, and the Kubernetes example
uses etcd.

The topology service exists for several reasons:

- It enables tablets to coordinate among themselves as a cluster.
- It enables Vitess to discover tablets, so it knows where to route queries.
- It stores Vitess configuration provided by the database administrator that is
  needed by many different servers in the cluster, and that must persist between
  server restarts.

A Vitess cluster has one global topology service, and a local topology service
in each cell. Since *cluster* is an overloaded term, and one Vitess cluster is
distinguished from another by the fact that each has its own global topology
service, we refer to each Vitess cluster as a **toposphere**.

### Global Topology

The global topology stores Vitess-wide data that does not change frequently.
Specifically, it contains data about keyspaces and shards as well as the
master tablet alias for each shard.

The global topology is used for some operations, including reparenting and
resharding. By design, the global topology server is not used a lot.

In order to survive any single cell going down, the global topology service
should have nodes in multiple cells, with enough to maintain quorum in the
event of a cell failure.

### Local Topology

Each local topology contains information related to its own cell.
Specifically, it contains data about tablets in the cell, the keyspace graph
for that cell, and the replication graph for that cell.

The local topology service must be available for Vitess to discover tablets
and adjust routing as tablets come and go. However, no calls to the topology
service are made in the critical path of serving a query at steady state.
That means queries are still served during temporary unavailability of topology.

## Cell (Data Center)

A *cell* is a group of servers and network infrastructure collocated in an area,
and isolated from failures in other cells. It is typically either a full data
center or a subset of a data center, sometimes called a *zone* or *availability zone*.
Vitess gracefully handles cell-level failures, such as when a cell is cut off the network.

Each cell in a Vitess implementation has a local topology service,
which is hosted in that cell. The topology service contains most of the
information about the Vitess tablets in its cell.
This enables a cell to be taken down and rebuilt as a unit.

Vitess limits cross-cell traffic for both data and metadata.
While it may be useful to also have the ability to route read traffic to
individual cells, Vitess currently serves reads only from the local cell.
Writes will go cross-cell when necessary, to wherever the master for that shard
resides.

Contact: vitess@googlegroups.com  **·**  
Announcements  **·**  
© 2017 Vitess powered by Google Inc
